# Annual acknowledgement of manuscript reviewers

**DOI:** 10.1186/s40104-016-0073-0

**Published:** 2016-02-26

**Authors:** Defa Li

**Affiliations:** China Agriculture University, Beijing, China

## Abstract

The *Journal of Animal Science and Biotechnology* editorial team would sincerely like to thank all of our reviewers who contributed to peer review for the journal in Volume 6 (2015).

**Satoshi Akagi**

Japan

**Toyoko Akiyama**

Japan

**Tracy Anthony**

USA

**MEOA Assumpção**

Brazil

**Fuller Bazer**

USA

**Donald Beitz**

USA

**Alvaro Belenguer**

Spain

**Francois Blachier**

France

**John Black**

Australia

**Walter Bottje**

USA

**Gaelle Boudry**

France

**Dianna Bourassa**

USA

**M. Bozkurt**

Turkey

**BJ Bradford**

USA

**ARJ Cabrita**

Portugal

**Fidel Ovidio Castro**

Chile

**Mirko Cattani**

Italy

**Sandra Cecconi**

Italy

**Fabrizio Ceciliani**

Italy

**AV Chaves**

Australia

**Johanne Chiquette**

Canada

**CL Chiu**

Australia

**Agata Chmurzyńska**

Poland

**John Church**

Canada

**KP Coffey**

USA

**R D’inca**

Czech Republic

**Teresa Davis**

USA

**Matthew H Deighton**

Australia

**Haiteng Deng**

China

**M Denli**

Spain

**Udaya Desilva**

USA

**Claudia Domini**

Argentina

**Michael ER Dugan**

Canada

**Ted Elsasser**

USA

**Vivek Fellner**

USA

**Catherine Field**

Canada

**Nathalie Le Floc'h**

France

**Niamh Forde**

United Kingdom

**Abigail Fowden**

United Kingdom

**Rafael Frias**

Finland

**Nicholas Gabler**

USA

**Dorian Garrick**

USA

**Ilias Giannenas**

Greece

**Jennifer Hernandez Gifford**

USA

**Enric Gisbert**

Spain

**Liara Gonzalez**

USA

**Robert Goodband**

USA

**JL Gordon**

Canada

**AR Greenhill**

Australia

**JP Gutiérrez**

USA

**Judith Hall**

United Kingdom

**Jae Yong Han**

Republic of Korea

**Steve Hart**

USA

**Pingli He**

China

**PM Heegaard**

Denmark

**Jung Min Heo**

Republic of Korea

**Francisco Hernández**

France

**Eric Van Heugten**

USA

**W Heuwieser**

Germany

**Charlotte Heyer**

Germany

**GM Hill**

USA

**Shinichi Hochi**

Japan

**Chien-An Hu**

USA

**Ryo Inoue**

Japan

**Robert Ivarie**

USA

**Joshua Jendza**

USA

**Younggeon Jin**

USA

**Wang Jinrong**

China

**Greg Johnson**

USA

**Henry Jorgensen**

Denmark

**Steve Kachman**

USA

**Mika Kahkonen**

Finland

**Filiz Karadas**

Turkey

**Yukinori Kazeto**

Japan

**Yoshiaki Kikkawa**

Japan

**Inho Kim**

Republic of Korea

**Sung Woo Kim**

USA

**F Klevenhusen**

Switzerland

**Terry Klopfenstein**

USA

**Robert A Knox**

USA

**Wilfried A Kues**

Germany

**G Laible**

New Zealand

**Jai-Wei Lee**

Taiwan

**Hong-Gu Lee**

Republic of Korea

**April Leytem**

USA

**Shengli Li**

China

**Robert W Li**

USA

**Hyun Lillehoj**

USA

**Hai Lin**

China

**Jan Erik Lindberg**

Sweden

**Jue Liu**

China

**Yanhong Liu**

USA

**Elena Llano**

Spain

**Juan J Loor**

USA

**F López-Gatius**

Spain

**Alberto Maria Luciano**

Italy

**Hailing Luo**

China

**Yuri M Lvov**

Germany

**Yongsheng Ma**

China

**Xi Ma**

China

**Elizabeth Maga**

USA

**Louisa G Mahaira**

Greece

**Subeer S Majumdar**

India

**L. Manning**

United Kingdom

**Edgar Garcia Manzanilla**

Spain

**Xiangbing Mao**

China

**José Martínez-González**

Spain

**D Martinez-Puig**

Spain

**Yutaka Masuda**

USA

**Mike McGrew**

United Kingdom

**Mark Mcguire**

USA

**Arcangelo Merla**

Italy

**Georgios Michailidis**

Greece

**Joris Michiels**

Belgium

**Yoshinori Mine**

Canada

**Nicolas Montalbetti**

Switzerland

**Hugo H Montaldo**

Mexico

**Robert J Moore**

Australia

**Francois Morle**

France

**Gota Morota**

USA

**JM Morrell**

Sweden

**Kasey Moyes**

USA

**V Muchenje**

South Africa

**Stefanie Muff**

Swaziland

**K Nagaoka**

Japan

**Margaret C Neville**

USA

**Pin Nie**

China

**Heiner Niemann**

Germany

**Martin Nyachoti**

Canada

**Bjorn Oback**

New Zealand

**Jack Odle**

USA

**Padraig O'Kiely**

Ireland

**Ana Oliveira**

Portugal

**Toshinori Omi**

Japan

**Christopher O'Neill**

Australia

**J Opiela**

Poland

**William R Otto**

United Kingdom

**Fred Owens**

USA

**G Paci**

Italy

**John Patience**

USA

**AK Patra**

India

**AF Pereira**

Brazil

**James Pettigrew**

USA

**C Pomar**

Canada

**EN Ponnampalam**

Australia

**Howard Prentice**

USA

**Roger Purchas**

New Zealand

**Shiyan Qiao**

China

**Ning Qu**

Japan

**Gerardo Quiroz-Rocha**

Mexico

**K. Ropka-Molik**

Poland

**Florence Rouleux-Bonnin**

France

**Luis A Rubio**

Spain

**Pedro Rubio**

Spain

**C. Sañudo**

Spain

**James Sartin**

USA

**G Scaglia**

USA

**Tom Scott**

Canada

**A Sechman**

Poland

**Marília Seelaender**

Brazil

**Asshan Shan**

China

**Yanbin Shen**

USA

**Paul Siciliano**

USA

**R Simões**

Brazil

**Jean Sirois**

Canada

**WF Skrzypczak**

Poland

**Diane Spurlock**

USA

**Hans Stein**

USA

**Helena Storchova**

Czech Republic

**Kunio Sugahara**

Japan

**Jorge Sztein**

Spain

**G Tellez**

USA

**WW Thatcher**

USA

**James A Thomson**

USA

**Nuria Tous**

Spain

**Annabelle Troegeler-Meynadier**

France

**Pedro Urriola**

USA

**Asier Vallejo**

Spain

**Francisca Velkers**

Netherlands

**Martin Verstegen**

Netherlands

**Marco Aurélio Vinolo**

Brazil

**Kimberly Vonnahme**

USA

**Elizabeth Walker**

USA

**R John Wallace**

United Kingdom

**Rosemary Walzem**

USA

**Junjun Wang**

China

**Xiaoqiu Wang**

USA

**Guangshun Wang**

USA

**Xin Wang**

USA

**Malcolm Watford**

USA

**DC Wathes**

United Kingdom

**Matthew Webster**

Sweden

**Tania de Azevedo Weimer**

Brazil

**Mayara Weller**

Brazil

**C Bruce A Whitelaw**

United Kingdom

**W Widlak**

Poland

**Guoyao Wu**

USA

**M Yamazaki**

Japan

**Wenzhu Yang**

Canada

**Fang Yang**

USA

**Kai Yuan**

USA

**Kai Yuan**

USA

**Kai Yuan**

USA

**Michael Zasloff**

USA

**Lidan Zhao**

USA

**Ruqian Zhao**

China

**Shien Zhu**

China

**Krzysztof Zyla**

Poland

